# Phospholipase A2 enzyme from the venom of Egyptian honey bee *Apis mellifera lamarckii* with anti-platelet aggregation and anti-coagulation activities

**DOI:** 10.1186/s43141-020-00112-z

**Published:** 2021-01-14

**Authors:** Doaa A. Darwish, Hassan M. M. Masoud, Mohamed M. Abdel-Monsef, Mohamed S. Helmy, Hind A. Zidan, Mahmoud A. Ibrahim

**Affiliations:** 1 Molecular Biology Department, National Research Centre, 33 El Bohouth St. (former El Tahrir St.), Dokki, P.O. 12622, Giza, Egypt; 2 Plant Protection Research Institute, Agricultural Research Center, Giza, Egypt

**Keywords:** Bee venom, Phospholipase A2, Purification and characterization, Anti-platelet aggregation, Anti-coagulation

## Abstract

**Background:**

Honey bee venom contains various enzymes with wide medical and pharmaceutical applications.

**Results:**

The phospholipase A2 (PLA2) has been apparently purified from the venom of Egyptian honey bee (*Apis mellifera lamarckii*) 8.9-fold to a very high specific activity of 6033 U/mg protein using DEAE–cellulose and Sephacryl S-300 columns. The purified bee venom PLA2 is monomeric 16 kDa protein and has isoelectric point (*p*I) of 5.9. The optimal activity of bee venom PLA2 was attained at pH 8 and 45 °C. Cu^2+^_,_ Ni^2+^, Fe^2+^_,_ Ca^2+^, and Co^2+^ exhibited a complete activating effect on it, while Zn^2+^, Mn^2+^, NaN_3_, PMSF, N-Methylmaleimide, and EDTA have inhibitory effect.

**Conclusions:**

The purified bee venom PLA2 exhibited anti-platelet aggregation and anti-coagulation activities which makes it promising agent for developing novel anti-clot formation drugs in future.

## Background

Honey bee venom contains a mixture of various energetic ingredients like enzymes, polypeptides, amino acids, amines and lipids that cause local inflammations and acting as anti-coagulators and analgesics [1–3]. Apitherapy is a medication type that utilizes honey bee components like honey, pollen, royal jelly, propolis, and venom (apitoxins) to treat many human diseases. It is achieved either directly by stings of bees or indirectly through extracting the bee venom and injecting bodies with it [4]. One of the most important bee venom enzymes is phospholipase A2 (PLA2). It is a lipolytic enzyme that hydrolyzes phospholipids at sn-2-acyl linkage to liberate free fatty acids and lysophospholipids [5–7]. PLA2 is the most fatal honey bee venom ingredient that composed of individual 128 amino acids polypeptide chain of four disulfide linkages. It works as an allergen and collaborate with different components defending the colony from predator and intruder animals [4, 8]. PLA2s were found in many sources such as mammalian pancreas, reptile venoms, insect venoms, and synovial fluids [9]. PLA2s can be classified into secretory (sPLA2), cytosolic Ca^2+^-dependent (cPLA2), and cytosolic Ca^2+^-independent (iPLA2) based on their properties [10]. PLA2s of bee, lizard and scorpion venoms are all secretory and Ca^2+^-dependent type [7, 11, 12]. PLA2 has broad variations of pharmacological characteristics including anti-human immunodeficiency virus (HIV), neurotoxicity, myo-toxicity, and neurites outgrowth inductions [13]. PLA2s have important functions in the cellular operations comprising digestion and metabolism of phospholipids, host defenses, atherosclerosis, signal transduction processes, membrane remodeling, and delaying oxidant-induced cell death [14, 15]. PLA2s are also connected with many human troubles like rheumatoid arthritis, autoimmune uveitis, respiratory distress syndrome, myocardial infarctions, and endotoxic shocks [16]. PLA2 can be used as a pharmacological factor for Alzheimer’s disease by enhancing α-secretase-dependent amyloid precursor protein processing to regulate membrane fluidity [17, 18]. PLA2 can also exert protective effects on airway inflammation in asthma [19]. For all these broad medical and pharmacological uses of PLA2, this study reports the isolation and biochemical characterization of PLA2 from the venom of Egyptian honey bee *Apis mellifera lamarckii*.

## Methods

### Venom collection

Honey bees colonies (*Apis mellifera lamarckii*) were obtained from Assiut Governorate, Egypt. Bee venom was extracted from 500 forager workers that were caught at entry of the colony and immobilized via rapid freezing at – 20 °C. Individuals were dissected, sting devices and venom reservoirs were removed, disrupted in tube with 2.5 ml dH_2_O, and finally centrifuged at 12000×*g* for 5 min at 4 °C and supernatant was obtained as crude venom.

### Chemicals

Phosphatydylcholine, Triton X-100, phenol red, Dithiothreitol (DTT), Phenyl methyl sulfonyl fluoride (PMSF), 1,10 Phenanthroline, bovine serum albumin (BSA), Diethylaminoethyl cellulose (DEAE-Cellulose), marker proteins, Sephacryl S-300, and thromboplastin were from Sigma Chemical (St. Louis, USA). The other chemicals were of analytical grade. Human blood samples were obtained from the laboratory of medical center hospital of our institute.

### Assay of phospholipase A2 enzyme activity

The reaction mixture of PLA2 activity assay consists of 2.5 ml 7.5 μmol Tris/HCl, pH 7.9 containing phosphatydylcholine (15 μmol), Triton X-100 (18 μmol), CaCl_2_ (5 μmol), and phenol red (80 μmol). The optical density was first recorded at 558 nm as a blank for each sample. Start the reaction by adding the enzyme solution, incubate for an hour at 37 °C, and then record the decrease in absorbance at 558 nm. One unit PLA2 activity is the amount of enzyme needed to hydrolyze 1 μmol phosphatidylcholine per hour at 37 °C [20].

### Purification of phospholipase A2 enzyme from honey bee venom

All experiments were performed at 4 °C. The crude (*Apis mellifera lamarckii*) venom extract was loaded on DEAE cellulose column (6× 2.4 cm i.d.) formerly equilibrated with 0.02 M Tris/HCI buffer, pH 7.8. Venom components were eluted with equilibration buffer containing NaCl gradients (0–1 M) with collection of 5 ml fractions. Fractions were monitored for PLA2 activity at 558 nm and that exhibiting PLA2 activity were collected, lyophilized, and utilized for further purification steps. These concentrated fractions were further loaded on Sephacryl S-300 column (142 cm × 1.75 cm i.d.) earlier equilibrated with 0.02 M Tris/HCI buffer, pH 7.8 with collection of 2 ml fractions. Fractions exhibiting PLA2 activity were stored at – 20 °C and thereafter utilized for studying homogeneity and characteristics of the purified PLA2.

### Electrophoretic analysis

Homogeneity of bee venom PLA2 was monitored on 7% Native-PAGE [21], 12% SDS–PAGE [22, 23] and isoelectric focusing PAGE [24, 25]. Coomassie Brilliant Blue R-250 was utilized in staining the proteins.

### Protein determination

Protein contents were determined utilizing the dye binding assay procedure with use of albumin from bovine serum (BSA) as a standard [26].

### Anti-platelet aggregation activity

Blood specimens were collected in sodium citrate and centrifuged at 250×*g* for 15 min at 4 °C to separate platelet-rich plasma (PRP) and platelet-poor plasma (PPP) [27]. Assays were carried out by incubating 100 μL PRP for 5 min at 37 °C in a 96-well microtiter plate, and the contents were mixed for 5 s and O.D. was read at 540 nm for 5 min every 15 s. Thereafter, 100 μl PRP was incubated with equal amount of PLA2 or PBS for 5 min at 37 °C followed by adding 30 μM ADP. The platelets aggregation induced only by ADP was considered as 100% control and other induced aggregations were compared with it [28].

$$ \mathrm{Percent}\ \mathrm{of}\ \mathrm{A}\mathrm{ggregation}=\frac{\mathrm{A}540\ \mathrm{of}\ \mathrm{PRP}\ \mathrm{with}\mathrm{out}\ \mathrm{PLA}2\hbox{-} \mathrm{A}540\ \mathrm{of}\ \mathrm{PRP}\ \mathrm{with}\ \mathrm{PLA}2}{\mathrm{A}540\ \mathrm{of}\ \mathrm{PRP}\ \mathrm{with}\mathrm{out}\ \mathrm{PLA}2\hbox{-} \mathrm{A}540\ \mathrm{of}\ \mathrm{the}\ \mathrm{PPP}} $$

### Anti-coagulation activity

The prothrombin time (PT) measures the plasma coagulation time at 37 °C in existence of tissue thromboplastin-calcium mixture. Mix 50 μl bee venom PLA2 with 50 μl plasma, incubate for 6 min at 37 °C then add 100 μl pre-warmed calcium-thromboplastin solution at 37 °C for determining the clotting time [29, 30].

### Statistical analyses

Tests were performed in triplicates unless stated otherwise and statistical analyses were performed in calculating average arithmetic mean and standard error (S. E.) [31].

## Results

### Purification of phospholipase A2 from honey bee venom

**Fig. 1:**
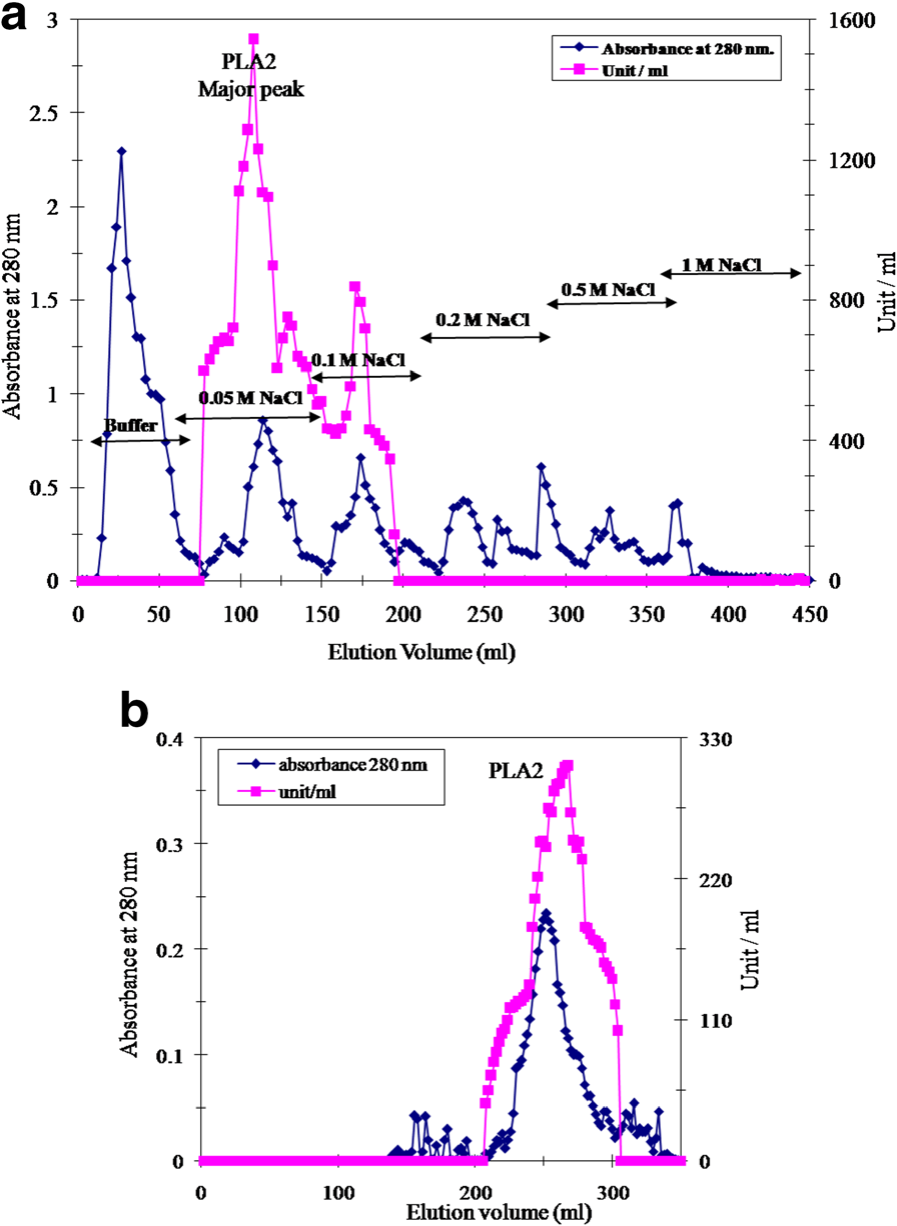
**a** DEAE-cellulose column chromatography elution profile of crude honey bee venom. **b** Sephacryl S-300 column elution profile of the DEAE-cellulose concentrates containing PLA2 activity

The purification of the PLA2 from the venom of the Egyptian honey bee (*Apis mellifera lamarckii*) was observed by PLA2 capability of hydrolyzing the phosphatidylcholine whereas the purification outlines exists in Table 1. The honey bee crude venom PLA2 specific activity was 675 units/mg protein. The bee venom PLA2 was eluted from the DEAE cellulose column as one large PLA2 peak with 0.05 M NaCl and a second small PLA2 peak eluted with 0.1 M NaCl (Fig. 1a). Honey bee venom PLA2 large peak was furthermore purified on Sephacryl S-300 column (Fig. 1b) that gave 6033 Umg^−1^ PLA2 represented 8.9-folds and 38% yield. A native bee venom PLA2 mass of 16-kDa was deduced via its elution volume from the size-exclusion column.

**Table 1:** A typical purification scheme of Egyptian honey bee venom PLA2

Purification steps	Total protein (mg)	Total activity (unit)	Specific activity	Yield (%)	Fold purification
Crude bee venom	47.2	31875	675	100	1
DEAE-cellulose major peak	9.3	20763	2234	65	3.3
Sephacryl S-300 fraction	2.0	12066	6033	38	8.9

### Electrophoretic analysis of purified honey bee venom phospholipase A2

**Fig. 2:**
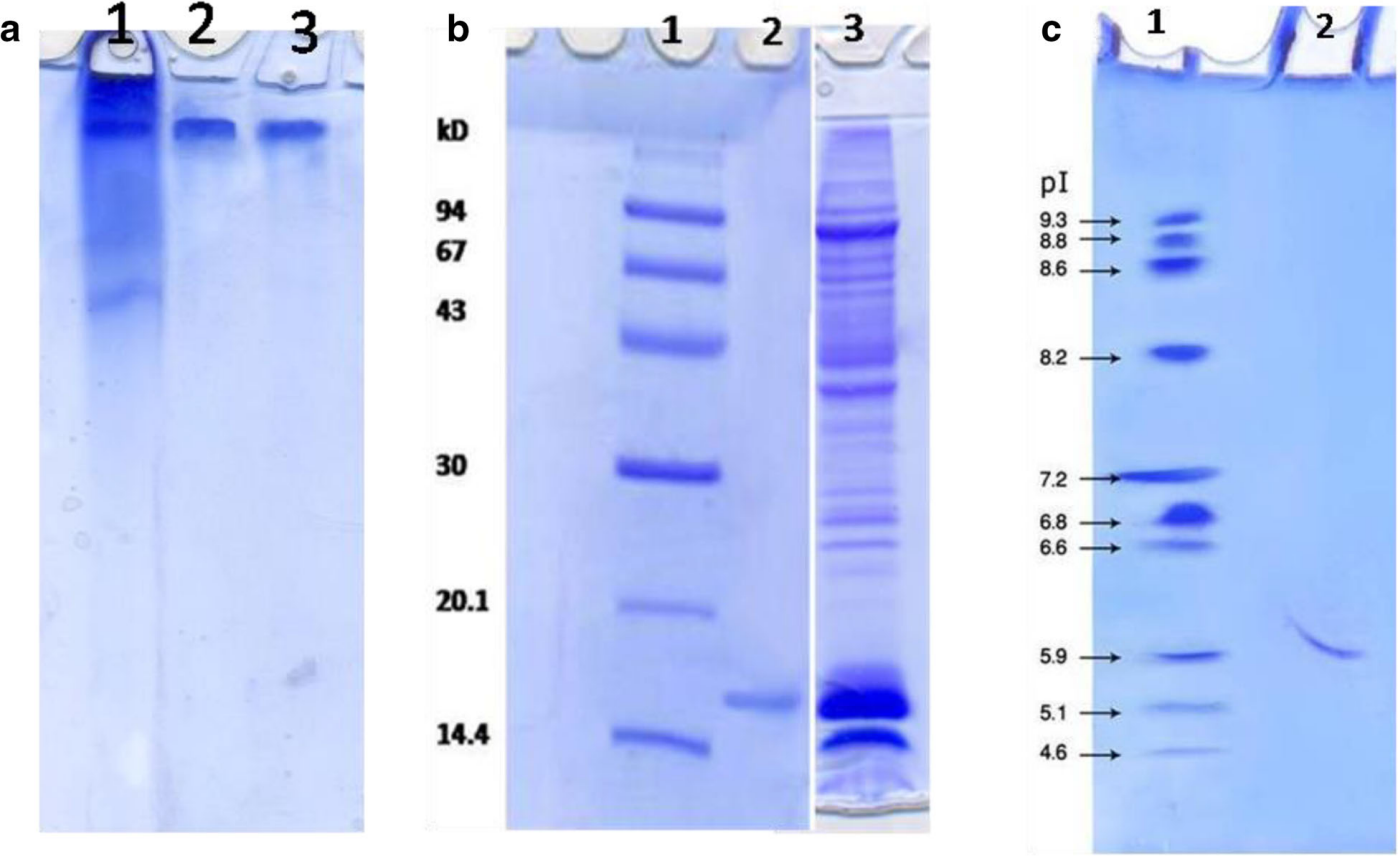
**a** Electrophoretic analysis of bee venom PLA2 different purification steps on 7% native PAGE: (1) crude bee venom, (2) DEAE-cellulose fraction, and (3) Sephacryl S-300 PLA2 fraction. **b** Bee venom PL A2 subunit on 12% SDS-PAGE: (1) molecular weight markers, (2) bee venom purified PLA2, and (3) crude bee venom. **c** Isoelectrofocusing: (1) isoelectric point (*p*I) marker proteins and (2) purified PLA2

Honey bee venom PLA2 enzyme various purification stages were visualized on 7% native-PAGE on which the purified PLA2 molecule was appeared as a singular protein band (Fig. 2a). On SDS-PAGE, honey bee venom PLA2 was detected as 16 kDa lone band (Fig. 2b) referring to one subunit molecule. The isoelectric point of PLA2 was estimated at pH 5.9 by isoelectric-focusing technique (Fig. 2c).

### Optimum pH, temperature, and *K*m value

**Fig. 3:**
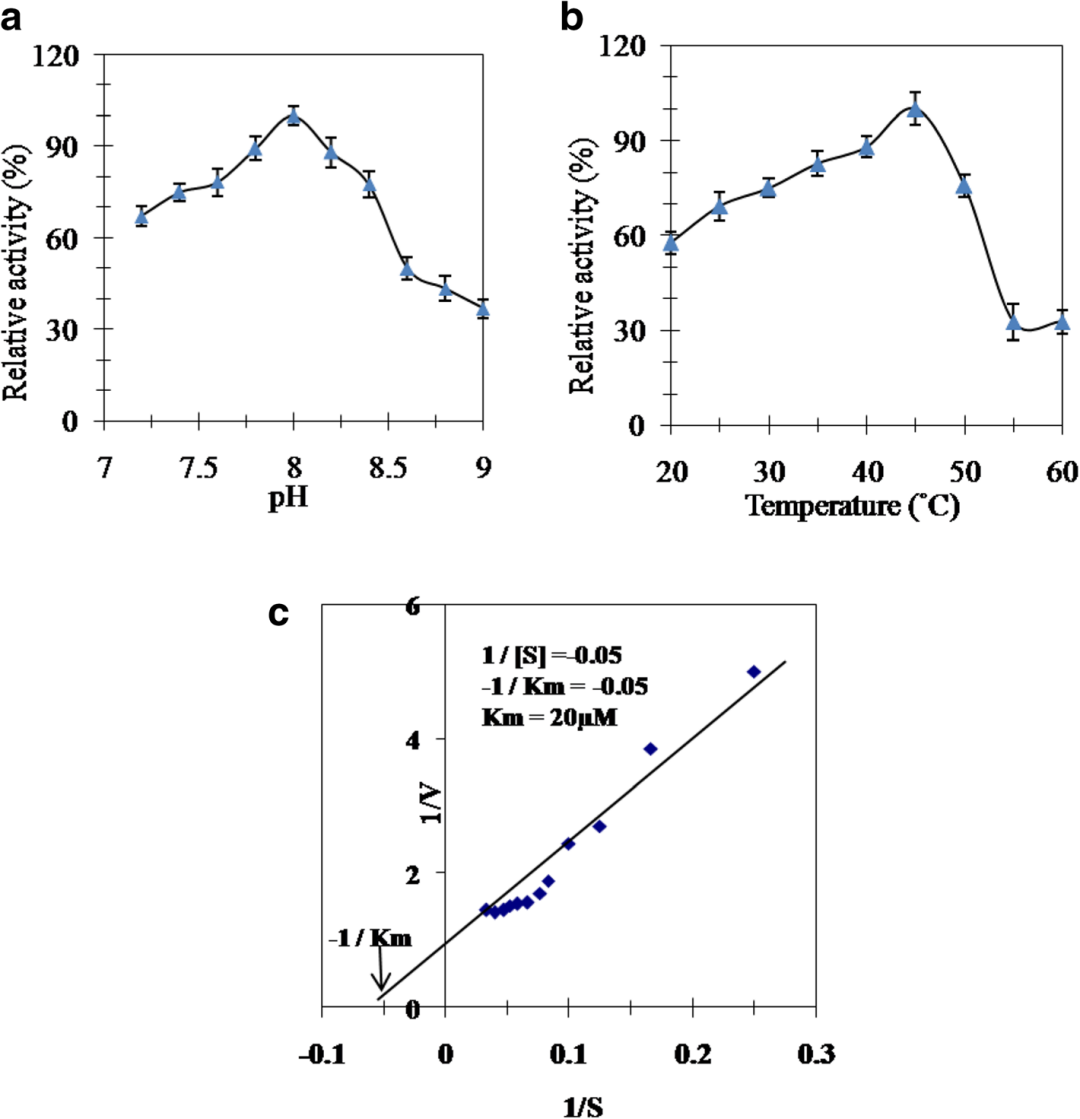
**a** Effect of pH on bee venom PLA2 using 0.02 M Tris-HCl buffer, pH (7.2–9.0). **b** Effect of temperature on bee venom PLA2. **c** Lineweaver-Burk plot relating the reciprocal of the reaction velocity of bee venom PLA2 to phosphatidylcholine concentrations

All of the tests were performed in triplicates unless stated otherwise. The effect of pH on the purified honey bee venom PLA2 was carried out utilizing Tris/HCl buffer, pH (7.2–9.0). The highest activity of bee venom PLA2 was recorded at pH 8.0 (Fig. 3a). The bee venom PLA2 was incubated at different temperatures (20–55 ^°^C) to know the suitable temperature for enzyme activity. Honey bee venom PLA2 enzyme showed its maximum activity at 45 °C (Fig. 3b). The *K*m value of bee venom PLA2 was calculated by Lineweaver-Burk plot as 20 μM phosphatidylcholine (Fig. 3c).

### Effect of cations and inhibitors

The purified bee venom PLA2 was incubated with two various concentrations of each divalent cation at 37 °C prior to its estimation assay. All Cu^2+^_,_ Ni^2+^, Fe^2+^_,_ Ca^2+^, and Co^2+^ raised PLA2 activity, while Zn^2+^ and Mn^2+^ lowered it (Table 2). Furthermore, we pre-incubated the purified bee venom PLA2 with several inhibitors for 5 min at 37 °C for calculation the inhibition percent in comparison with not inhibited control. All NaN_3_, PMSF, N-Methylmaleimide, and EDTA have inhibitory effect on bee venom PLA2 (Table 3).

**Table 2:** Effect of divalent cations on Egyptian honey bee venom PLA2

Reagent	Concentration (mM)	Residual activity (%)
Control	-----	100.0
CoCl_2_	5.0	128.6
	2.0	126.9
MnCl_2_	5.0	80.9
	2.0	88.6
FeCl_2_	5.0	296.4
	2.0	203.4
ZnCl_2_	5.0	70.1
	2.0	81.0
CuCl_2_	5.0	316.1
	2.0	274.3
NiCl_2_	5.0	205.5
	2.0	156.1
MgCl_2_	5.0	103.2
	2.0	100.7
CaCl_2_	5.0	185.4
	2.0	140.8

**Table 3:** Effect of various inhibitors on Egyptian honey bee venom PLA2

Reagent	Concentration (mM)	Inhibition %
Control	-----	0.0
β-Mercaptoethanol	5.0	27.5
1,10 Phenanthroline	5.0	18.8
NaN_3_	5.0	73.4
Iodoacetic acid	5.0	5.7
N-Methylmaleimide	5.0	37.4
DL-Dithiothreitol (DTT)	5.0	22.5
EDTA	5.0	33.2
Phenylmethylsulfonylfluoride (PMSF)	5.0	39.5

### Anti-platelet aggregation and anti-coagulation activities of PLA2

**Fig. 4:**
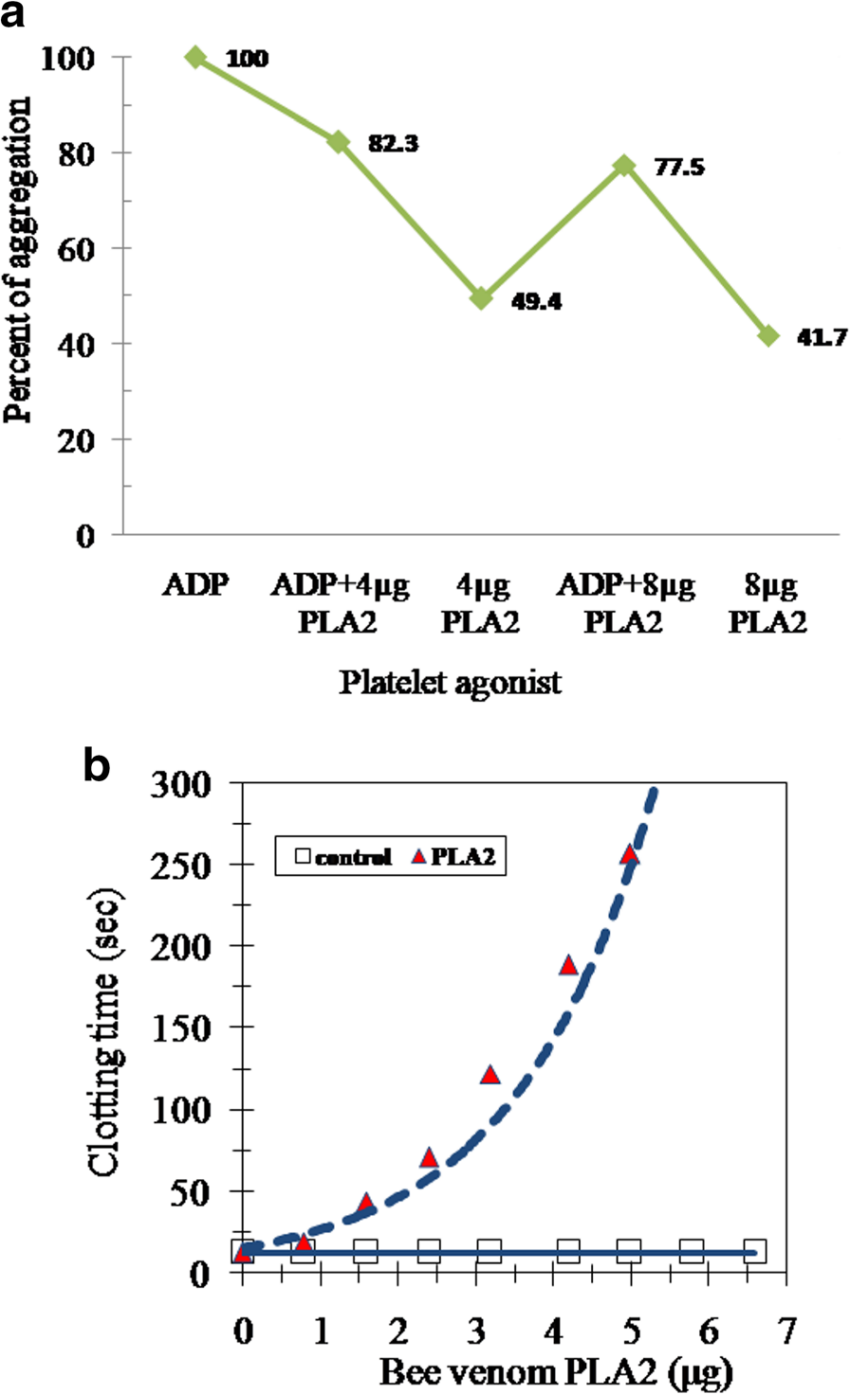
**a** Anti-platelet aggregation effect of bee venom PLA2. **b** The anti-clotting activity of bee venom PLA2

Eight micrograms of PLA2 purified from Egyptian honey bee *Apis mellifera lamarckii* venom prevented the aggregation of PRP by diminishing 60% of the ADP-stimulated platelets aggregation (Fig. 4a). Various concentrations of bee venom PLA2 were assayed for the inhibition of the extrinsic coagulation pathway. Bee venom PLA2 prolonged the PT time potently since 6 μg prevented the coagulation completely (Fig. 4b).

## Discussion

Bee venom components are important in pharmaceutical industry and drug formulations. The honey bee venom has high biological activity and is a better form for certain potential pharmacological sources [32]. Bee venom PLA2 was stated to have great effects as anti-inflammatory, anti-neuronal injury, anti-nociceptive, anti-tumor, anti-parasite, and anti-bacterial [17]. Honey bee venom PLA2 was isolated, purified, and characterized from various bee venom species [33, 34]. PLA2 was purified chromatographically on various matrixes including CM-Sephadex and Sephadex G-75 columns [35]; Q-Sepharose, S-Sepharose and C8 RP-HPLC [36]; **Mono-S Sepharose, Mono-Q Sepharose, and** RP-HPLC C-8 columns **[**11**]**; and Sephadex G-75 and Vydac C18 RP-HPLC columns [37]. In this study, PLA2 from Egyptian honey bee venom was purified by a simple and reproducible method consisting of two successive chromatographic runs. This procedure seemed to be valid to produce a homogenous PLA2 form. Fractionation of the crude honey bee venom on DEAE-cellulose column resulted in one major PLA2 form and another minor one (Fig. 1a). After gel filtration on Sephacryl S-300 column, an active PLA2 peak was eluted with 16 kDa native molecular weight (Fig. 1b). There was an increase in purification fold from 3.3 to 8.9 and specific activity from 2234 to 6033 units/mg protein with 38% recovery (Table 1). This finding is similar to that of Sallau et al. [38], who demonstrated that the increase in purification folds and specific activity of PLA2 was attributed to the removal of other synergistic interacting constituents of the venom. With consideration to the neglected DEAE-cellulose PLA2 minor peak, the considerable acquired bee venom PLA2 yield could refer to the suitability of the purification procedure used for this enzyme production. *Echis ocellatus* venom PLA2 was 43% yielded which represented 16-folds purification [39]. Egyptian Bee venom PLA2 homogeneity was indicated by single band on native PAGE (Fig. 2a). Bee venom PLA2 mass was estimated by SDS-PAGE as 16 kDa indicating the monomeric structure of the enzyme (Fig. 2b). *Apis mellifera caucasica* bee venom PLA2 was stated as 14 kDa [40], European honey bee *Apis mellifera* PLA2 was reported to have three forms of 16, 18, and 20 kDa [41] and Iranian bees PLA2 was reported to had two forms of 15 and 20 kDa [3]. The isoelectric point of Egyptian bee venom PLA2 was estimated at 5.9 (Fig. 2c), which is lower than the Carniolan subspecies venom PLA2 *pI* at 7.05 [42] and European honey bee (*Apis mellifica*) *pI* at 10.5 (Shipolini et al. 1971). Most *hymenopterous* venoms PLA2 *pI* ranging from pH 9 to 12 [43], while *Bothrops leucurus* snake venom PLA2 *pI* at 5.4 [44]. The Egyptian bee venom PLA2 displayed its optimum activity at pH 8 (Fig. 3a) similar to Turkey pancreatic PLA2 [12]. The Egyptian bee venom PLA2 attained its highest activity at 45 °C (Fig. 3b) that agreed with other findings [11]. Similar to the findings of Sallau et al. [38] and Ibrahim et al. [39], low *K*m value of 20 μM phosphatidylcholine (Fig. 3c) was estimated for Egyptian bee venom PLA2 indicating a high catalytic affinity of the enzyme toward phosphatidylcholine. The PLA2 activity was increased in the presence of Cu^2+^_,_ Ni^2+^, Fe^2+^_,_ Ca^2+^, and Co^2+^. On the other hand, Zn^2+^ and Mn^2+^ partially inhibited PLA2 (Table 2). Zinc, barium, and manganese ions have inhibitory effect on snake venom PLA2 (*Crotalus damanteus*) while calcium ion acted as enhancer effect on that of cobra venom [45]. Egyptian bee venom PLA2 activity was inhibited by β-Mercaptoethanol, DL-Dithiothreitol, N-Methylmaleimide, and 1,10 Phenanthroline affirming the role of thiol groups in PLA2 effectiveness. The existence of serine residue in PLA2 active site was affirmed by its inhibition by PMSF, while the metallo-enzyme nature of the molecule was indicated via PLA2 inhibition with EDTA (Table 3). PLA2 from Egyptian honey bee was effective in delaying the blood clotting and platelet aggregation. Bee venom PLA2 prevented the aggregation of blood platelets when tested against PRP in comparison with ADP-stimulated platelets aggregation (Fig. 4a). Also, bee venom PLA2 showed anti-coagulation effect, where the presence of purified bee venom PLA2 prolonged the prothrombin time gradually by increasing the enzyme concentration (Fig. 4b).

## Conclusion

In conclusion, the main achievement of this study is the preparation of homogenous PLA2 from Egyptian bee venom by a straightforward purification procedure. Honey bee venom PLA2 could have a role in treating variety of diseases as anti-platelets aggregation and anticoagulant agent. Finally, the current study provides the bee venom PLA2 as a promising agent for developing novel anti-clot formation drugs in future.

## Data Availability

All data and materials are available.

## Abbreviations

PLA2: Phospholipase A2
dH2O: Distilled water
DEAE–cellulose: Dietheylaminoethyl cellulose
PMSF: Phenylmethylsulfonyl flouride
BSA: Bovine serum albumin
PAGE: Polyacrylamide gel electrophoresis
ADP: Adinosine diphosphate
PRP: Platelet-rich plasma
PPP: Platelet-poor plasma
